# Genetic population structure and differentiation of Western Iranian Oxynoemacheilus *argyrogramma* (Heckel, 1847) using SSR markers 

**Published:** 2014-09

**Authors:** Hamed Kolangi-Miandare, Ghasem Askari

**Affiliations:** 1 Department of Fisheries Sciences, Gorgan University of Agricultural Sciences and Natural Resources, Iran; 2 Aquatic Ecology, Gorgan University of Agricultural Sciences and Natural Resources, Iran

**Keywords:** *Oxynoemacheilus argyrogramma*, Genetic diversity, Microsatellite, Iran

## Abstract

This study was carried out to investigate the genetic diversity and population structure of 90 specimens of *Oxynoemacheilus argyrogramma *collected from Sepidbarg, Gamasiab and Ghaleji rivers, in the west of Iran. Analyses using three microsatellite loci indicated that the average number of alleles in the population was 12, which was well above the reported values for freshwater fishes. The expected (He) and observed (Ho) heterozygosity means were 0.865 and 0.576, respectively. Almost all loci showed deviation from the Hardy-Weinberg equilibrium (HWE). The results demonstrated that *Oxynoemacheilus argyrogramma* had desirable genetic diversity in the investigated regions.

## INTRODUCTION

Confirmed freshwater fishes of Iran comprise of 202 species in 104 genera, 28 families, 17 orders and 3 classes. The dominant order is Cypriniformes with 120 species comprising 59.4% of the fauna, followed by Perciformes (28species, 13.9%), Cyprinodontiformes (10 species, 5.0%) and Clupeiformes (9 species, 4.5%). The most diverse family is the Cyprinidae with 93 confirmed species (46.0%) followed by Gobiidae with 22 species (10.9%), Nemacheilidae with 22 species (10.9%), Clupeidae with 9 species (4.5%), Cyprinodontidae with 8 species (3.9%) and Salmonidae with 7 species (3.5%) [1, 2]. The *Oxynoemacheilus argyrogramma* species (Heckel, 1847) belongs to the Nemacheilidae family stone loaches of the West of Iran. Like most river loaches of the Nemacheilidae family, these fish are small benthic species that live in swift-flowing water, mainly on gravel or stony substrata [3]. River loaches usually inhabit river systems and have little migration habits; therefore, they are ideal bio indicators for the study of freshwater fauna biogeography [4].

 Genetic diversity enabls environmental adaptation can assure survival chances of one species or population and is considered essential for the long-term survival of species [5]. Microsatellite markers are important tools for the study of molecular phylogeography and population genetics because of the advantages of high polymorphism, ease of genotyping and co-dominant inheritance [6]. Microsatellite DNA markers or simple sequence repeats (SSRs) are tandem repeated motifs of 1-6 bases found in all prokaryotic and eukaryotic genomes utilized in the assessment of genetic variation and population differentiation studies for a variety of vertebrates [7, 8]. In the present study, the genetic differentiation of diverse *Oxynoemacheilus* *argyrogramma* populations from three different river systems was examined using SSR markers. Three polymorphic microsatellite markers were developed to assess the genetic breeding of *Oxynoemacheilus argyrogramma*.

## MATERIALS AND METHODS

**Figure 1:**
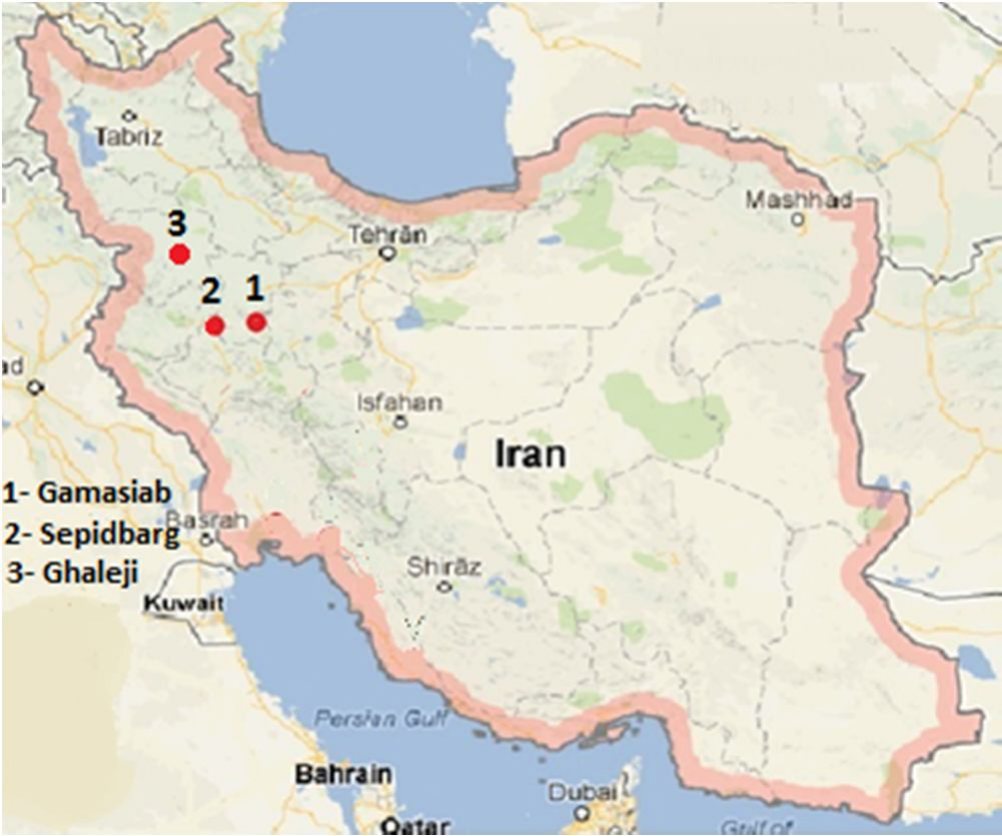
The sampling region for the *Oxynoemacheilus* *argyrogramma* (1: Gamasiab; 2: Sepidbarg; 3: Ghaleji)

**Sampling** **and DNA extraction: **Analyses were carried out based on 90 collected specimens from Sepidbarg (34ᵒ 52^/^ 17^//^ N; 46ᵒ 20^/^ 59^//^ E), Gamasiab, Kermanshah Province (34ᵒ 15^/^ 36^//^ N; 47ᵒ 23^/^ 44^//^ E) and Ghaleji, Kurdistan Province (35ᵒ 51/ 18// N; 45ᵒ 47^/^ 18^//^ E) rivers (Fig. 1). Collected samples were preserved in 96% ethanol until used. DNA was isolated by the phenol–chloroform procedure [9]. The quality and quantity of DNA were assessed by agarose gel (1%) electrophoresis and spectrophotometry. The extracted DNA was then stored at 4 °C for further analysis.

**SSR marker amplification:** Three primer pairs for SSR markers, Bbar5, Bbar8 and Bbar9 [10] were used (Table 1). Each reaction consisted of 50ng DNA template, 1.5 mM MgCl_2_, 0.2 μL forward and reverse primers, 0.2mM deoxyribonucleotide triphosphates (dNTP) and 0.5U *Taq* polymerase. Amplification conditions were as follows: a pre-denaturation for 3 min at 94°C, 35 cycles of 30 s at 94°C, 30 s at the selected higher annealing temperature, 30 s at 72°C, and 5 min at 72°C. Amplification products were separated by electrophoresis through 8% denaturing polyacrylamide gels. Detection of allele sizes obtained by the silver staining method was determined by comparing them to known DNA sequencing ladders.

**Table 1:** Detailed characteristics of amplified SSR loci in *Oxynoemacheilus argyrogramma*

**Locus**	**Primer sequence**	**Size (bp)**		**Annealing temp. (°C)**
**Bbar5**	F: ATAATCACAGCCCCGCAGAG R: GGGTGGTGGAATATATTGGAAA	84-120	12	55
**Bbar8**	F: GAGCAACAGCTGCTGTAGGA R: GTCGGACCAACCTGAAAACT	360-492	14	50
**Bbar9**	F: AATACGAAACTACTTGGTAATGGC R: GTGAAAAGGTCCAGTTAAAAGC	176-248	12	48

**Scoring and statistical analyses: **Sizes of individual alleles were determined in relation to a 50 bp DNA size standard using the GenePro Analysis software. GenAlex software package, version 6.5 [11], was used to calculate the frequency of alleles, as well as observed (H_o_) and expected (H_e_) heterozygosities and also to test for significant deviations from the Hardy-Weinberg equilibrium. Observed and expected genotype frequencies were then compared for each locus. The genetic distance between population pairs was estimated from the Nei standard genetic distance and the genetic similarity index [12]. Genetic differentiation between populations was evaluated calculating pairwise estimates of F_ST_ values.

## RESULTS AND DISCUSSION

All three loci (Bbar5, Bbar8 and Bbar9) were polymorphic in all populations of this study. The number of observed and effective alleles, observed heterozygosity, expected heterozygosity and fixation index are shown in Table 2. In the three *Oxynoemacheilus argyrogramma* populations, the number of effective alleles ranged from 4.84 to 14.23, and the average for each population was 8.3 for Sepidbarg (Se), 8.07 for Gamasiab (Ga) and 8.07 for Ghaleji (Gh). The observed alleles ranged from 6 to17, the average for each population being 13.66 (Se), 11.66 (Ga) and 10.66 (Gh). The expected heterozygosity range was from 0.793 to 0.930 and the average for each population was 0.8783 (Se), 0.874 (Ga) and 0.843 (Gh). The average observed heterozygosity of each population was 0.636 (Se), 0.667 (Ga) and 0.424 (Gh). The Bbar8 locus had the highest number of alleles (17) and the Bbar9 had the lowest (Table 2). 

For 3 of the 9 tests, significant deviations from the Hardy-Weinberg expectations (HWE) were detected (Table 2). Pair-wise F_ST_ values and genetic distances, calculated based on the reduced set of three microsatellite loci, are given in Table 3. The population differentiation (*F*ST) metric for the Sepidbarg and Gamasiab populations was 0.017, while for Sepidbarg-Ghaleji and Gamasiab-Ghaleji, it was found to be 0.045 (Table 3). The estimated gene flow (Nm) value between Sepidbarg-Gamasiab, Sepidbarg-Ghaleji and Gamasiab-Ghaleji were 14.03, 5.35 and 5.27, respectively, (Table 3). Analysis of the distribution of genetic variation indicated that variation was very high within the populations (96%) but low (4%) among them. The UPGMA dendrogram constructed on the basis of the DA distances showed only two major clusters (Fig. 3).

**Table 2:** Genetic variability of three microsatellite loci in three populations for *Oxynoemacheilus argyrogramma*

Location		Bbar5	Bbar8	Bbar9	Mean
	N_a_	12	16	13	13.66
	N_e_	7.68	9.77	7.44	8.30
Sepidbarg	H_o_	0.636	0.409	0.864	0.636
	H_e_	0.870	0.898	0.866	0.878
	F_IS_	0.268	0.544	0.002	0.272
	P_HW_	**	***	ns	-
	N_a_	12	12	11	11.66
	N_e_	9.58	6.96	7.68	8.07
Gamasiab	H_o_	0.727	0.500	0.773	0.667
	H_e_	0.896	0.856	0.870	0.874
	F_IS_	0.188	0.416	0.112	0.239
	P_HW_	ns	***	ns	-
	N_a_	9	17	6	10.66
	N_e_	5.14	14.23	4.84	8.07
Ghaleji	H_o_	0.682	0.318	0.273	0.424
	H_e_	0.806	0.930	0.793	0.843
	F_IS_	0.154	0.658	0.656	0.489
	P_HW_	***	***	***	-

**Notes:** Na, number of observed alleles; Ne, number of effective alleles; Ho, observed heterozygosity; He, expected heterozygosity; Fis, fixation indices; PHW, Hardy-Weinberg probability test

* P < 0.05,

** P < 0.01,

*** P < 0.001, n.s, non-significant

**Table 3:** Multilocus Nm (below diagonal) and F_ST_ values (above diagonal) between pairs of *Oxynoemacheilus* *argyrogramma* populations across all loci

	**Sepidbarg**	**Gamasiab**	**Ghaleji**
**Sepidbarg**	-	0.017	0.045
**Gamasiab**	14.03	-	0.045
**Ghaleji**	5.35	5.27	-

**Figure 2:**
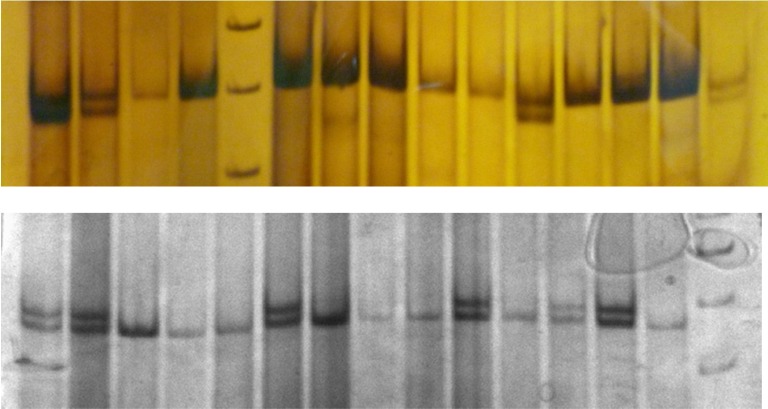
Microsatellite profiles of *Oxynoemacheilus argyrogramma* at loci Bbar5 and Bbar9

**Figure 3:**
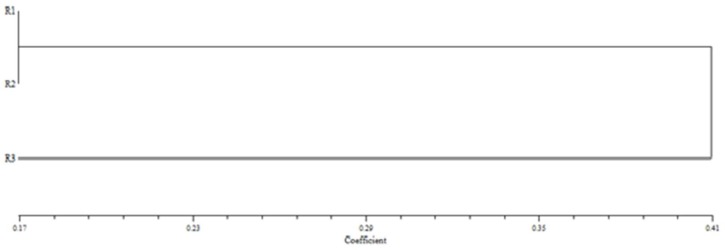
UPGMA dendrogram of *Oxynoemacheilus argyrogramma* populations based on a matrix of Genetic distance

Species adapt to various environments based on biological variation, which is one of the important factors for evaluating species' resources. A precondition to maintaining the highest level of genetic variation is making persistent use of a species' resources [13]. Heterozygosity serves as an indicator of evolutionary potential and is important in determining population dynamics as well as population viability [14]. The results of this study are consistent with earlier reports, suggesting the possibility of using primers interspecifically among teleost [15]. A precise estimation of population structure and genetic distances from microsatellite data is dependent on sample size, number of loci, number of alleles, and range in allele size [16].

Based on the findings of the present study, variation was high among the three *Oxynoemacheilus* *argyrogramma* populations (N_a_= 12, H_O_ = 0.576, H_E_ = 0.865). The results of the study were comparable in variability to those reported by DeWoody and Avise [17] regarding other freshwater fishes (Na: 9.1, He: 0.54), such as *Paraschistura bampurensis* (Na: 13, He: 0.872, [1]) and *Oxynoemacheilus kiabii* (Na: 811.5, He: 0.850, [18]) which have small populations and a high gene flow.

The Hardy-Weinberg disequilibrium is common in many fishes, but deviations to the equilibrium generally prevail over heterozygote deficits [1, 18, 19] resulting from factors involving reproductive systems, presence of null alleles, and a Wahlund effect (reduction of heterozygosity in a population caused by subpopulation structure). The average expected heterozygosity was highest in Gamasiab (0.667) and lowest in Ghaleji populations (0.424). The average observed heterozygosity values of all populations were lower than the corresponding expected heterozygosity values.

According to the expressions of Nm=(1−F_ST_)/4 F_ST_ [20], the Nm average between populations was 7.348. Theoretically, if the value of Nm is below 1, genetic drift is considered as the main factor of genetic differentiation, but if it is more than 1, gene flow is the main factor. The results of the present study revealed that migration of this species was the main reason for the genetic differentiation between the samples. 

Violations of the Hardy-Weinberg assumptions can cause deviations from expectation. Reduction in size of a population is considered to be one of the few factors that might be responsible for such deviations. Small population size causes a random change in genotypic frequencies, particularly if the population is very small due to genetic drift. Analysis of molecular variance (AMOVA) is a suitable criterion to assess population structure and to determine the differentiation and genetic similarity between populations [21]. According to the F_ST _index, the genetic diversity between the populations was 4% and the mean of the F_ST_ index was about 0.039, which represent the low differentiation between the three populations. According to Wright (1987) [22], an Fst value of less than 0.05 indicates low differentiation among communities.

Clustering order reflects relationships between populations. Using an UPGMA dendrogram, two separated communities were identified in these rivers. The genetic structure of *Oxynoemacheilus* *argyrogramma* in these rivers was probably caused by past migrations. To characterize and distinguish *Oxynoemacheilus* *argyrogramma* populations, microsatellite loci should be preferred because of their generally higher variability and better performance, particularly if populations are within geographical regions. Our study indicated that the three populations had moderate levels of polymorphism and genetic variation. This information should be taken into account for future genetic conservations and stock improvement plans.

## Notes

**Acknowledgments: **This work was supported by grant awarded to Dr. Hamed Kolangi Miandare and Gorgan University of Agricultural Sciences and Natural Resources. We deeply appreciate the respected reviewers for their valuable suggestions to improve the manuscript.
